# Inhibition of the *Naja naja* venom toxicity by polymeric nanoparticles loaded with *Leucas aspera* methanolic extract

**DOI:** 10.3389/fphar.2024.1385213

**Published:** 2024-05-09

**Authors:** Priyanka Singh, Gurunathan Jayaraman

**Affiliations:** School of Biosciences and Technology, Vellore Institute of Technology, Vellore, Tamil Nadu, India

**Keywords:** chitosan, *Leucas aspera* (Willd.), *Naja naja*, nanoparticle, controlled release

## Abstract

**Background:**

Snakebite is a neglected tropical disease that affects millions of people worldwide. Developing effective treatments can make a significant contribution to global health efforts and public health initiatives. To reduce mortality due to snakebite, there is an immediate need to explore novel and effective treatment methodologies. In that context, nanoparticle-based drug delivery is gaining a lot of attention. Hydrophilic nanoparticles are suitable for the delivery of therapeutic peptides, proteins, and antigens.

**Methods:**

The present investigation is aimed at evaluating the anti-ophidian potential of the methanolic extract of the ethno-medicinal herb *Leucas aspera* (Willd.) loaded within chitosan nanoparticles (CNP-LA), against the Indian cobra (*Naja naja*) venom enzymes. For this purpose, nanoparticles were prepared using the ionic gelation method to enhance the efficacy of the extract. The physicochemical and structural features of nanoparticles were investigated using dynamic light scattering (DLS), Fourier-transform Infrared (FTIR), field emission scanning electron microscopy (FE-SEM), and X-ray diffraction **(**XRD) techniques.

**Results:**

It was found that CNP-LA has an average size of 260 nm with a polydispersity index of 0.132 (PDI) and zeta potential of 34.7 mV, with an encapsulation efficiency of 92.46%. The *in vitro* release study was performed at pH 5.0 and 7.4. Furthermore, *in vitro* studies indicated that CNP-LA inhibited the phospholipase A2, hemolytic, and caseinolytic activities of *Naja naja* venom with the percentage inhibition of 92.5%, 83.9%, and 94.5%, respectively.

**Conclusion:**

This is the first report on the application of herbal methanolic extract loaded within chitosan nanoparticles for neutralizing snake venom enzymes with increased efficiency.

## Introduction

Snakebites are a significant public health issue in several parts of the world, especially in the regions of tropical and subtropical countries. According to the reports of WHO (World Health Organization), around 5.4 million snakebite cases occur every year, resulting in approximately 1.8 million envenomation and 138,000 deaths (WHO, 2019). However, these estimates are likely to be conservative due to underreporting and ineffective documentation. The distribution of snakebites varies geographically, with the highest numbers occurring in South Asia, Southeast Asia, and Sub-Saharan Africa, whereas, India has reported the maximum number of deaths occurring by snakebites in the world, with an estimated 58,000 deaths per year (Suraweera et al., 2020). The major snake species responsible for the maximum snakebite death cases in India is the Indian cobra (*Naja naja*) (Senji Laxme et al., 2021). Its venom consists of neurotoxin, and it produces systemic poisoning, thereby causing respiratory paralysis and death. It is considered a very dangerous snake among the species (Achyuthan and Ramachandran, 1981).

The genus *Leucas* belongs to the Lamiaceae family. The diverse pharmacological characteristics of several species in the genus *Leucas*, including those with antimicrobial, antioxidant, anti-inflammatory, cytotoxic, anticancer, antidiabetic, and wound-healing properties, have been the subject of in-depth research. As a result, the phytochemistry and pharmacological potential of the genus *Leucas* indicate that it has a promising potential as a significant source of natural compounds for drug development and discovery (Prajapati et al., 2010; Gopi et al., 2014; Hiremath et al., 2022).

The formulation of polymer-based nanoparticles has gained much attention in the field of therapeutics by increasing the efficiency of drugs via different routes such as intravenous, oral, and mucosal administration (Janes et al., 2001). Several polymer materials have been used for the formulation of nanoparticles, and among them, chitosan is reported to have more advantages for therapeutic applications. CS is a biodegradable polysaccharide known for its bioadhesive properties. Extensive research has demonstrated that CS is non-toxic and compatible with soft tissues (Bullock et al., 2000). Therefore, it has found widespread applications in pharmaceutical research and industry, serves as an efficient carrier for drug delivery, and is an effective material in biomedical applications (Mao et al., 2001). The primary amine groups contribute to the positive charge in chitosan and are important for mucoadhesive property, antimicrobial activity, and wound healing property that makes CS very useful in therapeutic applications (Berscht et al., 1994; Takeuchi et al., 1996; Mohammadpour dounighi et al., 2010).

The present study is aimed at investigating the efficiency of the methanolic extract of *L. aspera* (Willd.) loaded within chitosan nanoparticles against specific snake venom enzymes. To date, to the best of our knowledge, there is very little or no report on the use of the nanoparticle-loaded extract against snake venom enzymes. The present study provides an alternate option for the management of snakebites with enhanced efficiency.

## Materials and methods

### Materials

Deacetylated CS powder of medium molecular weight and medium viscosity (200–800 cP, ≥75%) was obtained from Sigma-Aldrich Chemicals Ltd. (USA). STPP was purchased from Sigma-Aldrich Chemicals. Leaves of *L. aspera* were collected from the nursery maintained by the Vellore Institute of Technology in October 2021 and were identified and authenticated by Dr. Siva R, (Botanist, Vellore Institute of Technology, Vellore, India). The voucher specimen (VITMN006-1) is maintained in the laboratory. All other materials and reagents used in this study are of analytical grade. Lyophilized snake venom (*Naja naja*) was purchased from Irula Snake Catcher’s Industrial Co-Operative Society Limited (Chennai, India). The venom was dissolved in 0.9% of Tris-HCl and centrifuged at 2,500 rpm for 10 min, and the supernatant was used for the study. The Indian snake antivenom was from VINS Bioproducts Limited.

### Extraction

Initially, the fresh plant leaves were shade-dried and powdered using a mortar and pestle using liquid nitrogen. Then, 350 g of shade-dried powdered leaves was soaked in 700 mL of methanol for 24 h and was kept constantly stirred in a shaker. The extraction was repeated three times by changing the solvent every 24 h. Furthermore, the extract was filtered using a Whatman filter paper to remove the impurities. Finally, the extract was concentrated using a rotary vacuum evaporator under reduced pressure at 40°C. The concentrated extract was further used for the estimation of phenolics, flavonoids, and formulation of nanoparticles.

## Determination of the total phenolic and total flavonoid concentrations in the LA extract

### Folin–Ciocalteu calorimetric assay

The estimation of the total phenolic content for the *L. aspera* methanolic extract was performed using the Folin–Ciocalteu colorimetric assay (Sánchez-Rangel et al., 2013). A measure of 500 µL of the extract containing 2% *L. aspera* extract was added and mixed thoroughly using 200 µL of the Folin–Ciocalteu phenolic reagent. Then, 2.5 mL of the 10% (w/v) Na₂CO₃ aqueous solution was added, and the solution mixture was incubated in the dark for 30 min. The absorbance was taken at 765 nm using a UV visible spectrophotometer. By using gallic acid as a standard, the total phenolic content was calculated and represented as mg of the gallic acid equivalent (GAE) per gram of the extract.

### Total flavonoid content

The estimation of the total flavonoid content for the *L. aspera* methanolic extract was performed using the aluminum chloride colorimetric method (Chang et al., 2002). In brief, 0.5 mL of the *L. aspera* extract and quercetin (standard) solution were added to 0.5 mL of 5% NaNO₂. Subsequently, 1 mL of 10% AlCl_3_ was added, and then, 2 mL of 1M NaOH was added. The solution mixture was incubated at room temperature for 30 min. Then, the absorbance was measured at 415 nm wavelength in a UV–visible spectrophotometer. A standard calibration curve was plotted with the known concentration of the quercetin standard solution. The total concentration of flavonoid in the extract was calculated and represented as mg of the quercetin equivalent per gram of the extract.

### Preparation of the *L. aspera* methanolic extract loaded in chitosan nanoparticles (CNP-LA)

To prepare CNP-LA, the ionic gelation method was used, as reported previously (Elzatahry and Eldin, 2008), with some modifications. In brief, 25 mg of chitosan powder was dissolved in 1% (w/v) of glacial acetic acid at pH 5.5 under continuous stirring at 500 rpm on a magnetic stirrer for 24 h. The CNP was prepared by the dropwise addition of 8 mL of the sodium tripolyphosphate (TPP) solution at different concentrations of 0.5%, 1%, and 2% (v/v). Then, 1%, 2%, 5%, and 10% of the LA extract solution was added to the TPP mixture, followed by the addition of 25 mL of the chitosan solution under continuous stirring (1 h at 1,100 rpm). Then, 4% (v/v) Tween 80 was added to the mixture and stirred for 15 min at 800 rpm. The chitosan suspension obtained was centrifuged (8,670 g, 30 min). Then, the pellet obtained was washed thrice with double distilled water. Subsequently, the pellet was re-suspended in double distilled water and ultra-sonicated at a frequency of 20 Hz for 15 min with the on and off pulse of 10 and 5 s, respectively (Sonics Vibrcell, Sonics & Materials, Inc., Newtown, CT, USA). The freshly prepared nanoparticles were filtered using syringe filters of 0.4 µm (Merck Millipore, Darmstadt, Germany) and lyophilized (Alpha 1–2 LSCbasic, Martin Christ, Germany) for further characterization. CNPs without the LA extract were prepared using the same procedure without the inclusion of LA.

## Characterization of CNP-LA

### UV-visible spectrophotometry

The absorption characteristics of CNP and CNP-LA (1 mL) were measured using a quartz cuvette of path length of 10 mm using a UV-visible spectrophotometer (JASCO V-670 PC) in the wavelength range of 200–800 nm.

### Dynamic light scattering

The particle sizes of CNP and CNP-LA were determined using a Zetasizer Nano series instrument (HORIBA Nano particle SZ-100, Japan). Lyophilized nanoparticles (CNP and CNP-LA) were re-suspended in distilled water (1:10 ratio) and sonicated for 15 min to hinder the aggregation of the particles, and then, the particle size for the nanoparticles was measured. The Zeta potential of CNP and CNP-LA was determined by measuring the electrophoretic mobility (UE) using a folded capillary cell with a Zetasizer Nano series instrument (HORIBA Nano particle SZ100, Japan).

### Fourier-transform infrared analysis

Functional groups of CNP and CNP-LA were analyzed by Fourier-transform infrared (FTIR) spectroscopy using a Spectrum RX FTIR spectrometer (IR Affinity-1, Shimadzu, Japan). For analysis, lyophilized powders of CNP and CNP-LA were mixed in the ratio of 2% w/w of potassium bromide. The mixture was ground into a very fine powder and compressed into a KBr disk under a hydraulic press at 10,000 psi. The spectrum was recorded in the range 4,000 cm^-1^ to 400 cm^-1^ with a resolution of 1 cm^-1^. Characteristic functional groups in the IR spectra were identified using the IRsolution version 1.60.

### Surface morphology (FESEM) and EDX (energy dispersive X-ray) analysis

The surface morphology of CNP and CNP-LA was examined by field emission scanning electron microscopy (FESEM) (Thermo Fisher FEI Quanta 250 FEG) operated at 30-kV high vacuum with 1.2 nm resolution. The sample was smeared on the glass slide (1 cm × 1 cm) and incubated overnight under vacuum to remove the water and other moisture content from the sample. Sputter coating was used to coat the slide with a thin film of gold sputter coating and analyzed under FESEM. Different characteristics and properties of the particles such as size, morphology, and structure were studied at different magnifications. EDX analysis was done for CNP and CNP-LA nanoparticles for the qualitative status of elements constituting nanoparticles.

### Powder X-ray diffraction (XRD) analysis

The crystalline nature of the lyophilized powder of CNP and CNP-LA samples was analyzed by X-ray diffraction (Burker D8 QUEST, Bruker AXS GmbH, Germany). Powder X-ray diffraction (Shimadzu, XRD 6000, Japan) was recorded in the range of 10°–80° using Cu–Kα radiation (1.5406 Å). FWHM (β) values and diffraction angles (q) were used to evaluate the morphology of the nanoparticles at a voltage of 45 kV and a current of 0.8 mA. A scanning range of 2θ/θ was selected, and a scanning speed of 10 min^-1^ was employed.

### Evaluation of encapsulation efficiency

The concentration of free LA was measured to evaluate the encapsulation efficiency of LA within the nanoparticles (Alqahtani et al., 2021). Then, 2 mL of CNP and CNP-LA dispersion was ultra-centrifuged (Hitachi WX Series, Hitachi Koki Co., Ltd., Tokyo, Japan) at 30,000 rpm for 30 min. The supernatant was collected, and the absorbance was measured at 270 nm of wavelength using a V-730 double beam UV–visible spectrophotometer (JASCO, Tokyo, Japan) to analyze the free *L. aspera* extract. Encapsulation efficiency (EE) was calculated using the subsequent formula mentioned below.

$$\text{EE }{\left(\%\right)}_{=}\frac{\left[\text{LA }\left(\text{Initial}\right)-\text{LA }\left(\text{free in the supernatent}\right)\right]\text{×100}}{\text{The initial amount of LA }}.$$

### The release of LA extract from CNP-LA (*in-vitro*)

As previously mentioned, to evaluate the release of metabolites from CNP-LA, a dialysis bag with a molecular weight cut off (10–12 kDa) was used (Sultan et al., 2022). A measure of 32 mg of each lyophilized formulation of CNP-LA was used in a dialysis bag in this method. The dialysis bags were then submerged in 50 mL of 1× PBS buffer at 7.4 and 5.0 pH at room temperature while constantly stirring at 1,000 rpm for 72 h. The sampling was done at predetermined periods by withdrawing 2 mL of the sample from the dialysate, and an equivalent amount of fresh buffer was added to maintain the total volume. The CNP-LA absorbance was measured using a V-730 double-beam UV-visible spectrophotometer (JASCO, Tokyo, Japan) at a wavelength of 270 nm. The percentage release of CNP-LA was calculated by plotting a graph OD *versus* the loaded CNP-LA concentration.

$$\text{LA released }{\left(\%\right)}_{=}\frac{\left(\text{Amount}\;\text{of}\;\text{LA}\;\text{released}\right)\times 100}{\text{Total}\;\text{weight}\;\text{of}\;\text{encapsulated}\;\text{LA}\;}.$$

## Snake venom inhibition studies

### PLA₂ inhibition assay

The enzymatic activity of PLA₂ of the venom was assessed using the egg yolk suspension method with slight modifications (Salama et al., 2018). In brief, 2 mL of egg yolk was added to the 1X PBS solution to prepare 2% (v/v) egg yolk suspension for the assay. A measure of 50 µg of venom was added to the egg yolk suspension, and the mixture was incubated at 37°C for 1 h. The PLA₂ activity was inferred by taking the absorbance at 900 nm of wavelength for the reaction mixture (V-730 double-beam UV-visible spectrophotometer, JASCO, Tokyo, Japan). For studying the inhibitory effect of nanoparticles over the venom, the venom was pre-incubated with various concentrations of CNP and CNP-LA (50 μL–250 µL) for 1 h at 37°C, before adding the substrate with antivenom (35 mg/mL). Antivenom (35 mg/mL) was used as a qualitative standard.

### Hemolytic assay

The hemolytic activity of the venom was determined using the RBC suspension method (Salama et al., 2018). Accordingly, citrate-added human blood collected in the AcCuvet Clot Activator tube was centrifuged at 3,000 rpm for 10 min at 4°C, and the plasma was discarded carefully. Then, 2% RBC (v/v) was suspended in 0.9 (w/v) of saline. To 100 µL of suspension, venom (10 mg/mL) was added and incubated at 37°C for 1 h. The experiment was terminated by the addition of ice-cold saline, followed by centrifugation at 3,000 rpm for 10 min (4°C). The release of the heme complex was detected by measuring the absorption of the supernatant at 490 nm (UV-vis spectrophotometer). For studying the inhibitory effect of nanoparticles over venom, the venom was pre-incubated with different concentrations of CNP-LA (50 mg/mL–250 mg/mL) and the standard AV (35 mg/mL) for 1 h at 37°C, before mixing the substrate.

### Caseinolytic activity

Azocasein was used as a substrate to study the caseinolytic activity of the venom using the protocol, as described previously (Munekiyo and Mackessy, 1998; Vera-palacios et al., 2022). The solution was prepared using 40 mg of azocasein diluted in 8 mL of Tris-HCl buffer (50 mM, pH 7.8). A measure of 10 μL of venom (100 mg/mL) was mixed with 90 µL of the substrate solution and 20 µL of the extract for inhibition studies. The solution was incubated for 1 h at 37°C, and then, 200 µL of 5% TCA (>99.0% Sigma) was added to all samples and subsequently centrifuged at 8,000 rpm for 5 min. Later, 150 µL of the supernatant was taken and placed in a 96-well micro-plate and mixed with 150 µL of 0.5M NaOH. The absorbance was measured using a plate reader (Bio-Rad xMark™, USA) at 450 nm. Enzyme activity was determined by comparing the corrected absorbance values. The standard AV (35 mg/mL) was incubated for 1 h at 37°C, before mixing the substrate.

### Statistical analysis

All the experiments were performed in triplicates using the proper controls, as specified. OriginPro was used to plot the data which were expressed as mean ± standard deviation, and a one-way analysis of variance (ANOVA) was used for the analysis of data. Based on the *p*-values, the results were evaluated at three levels of statistical significance (*p* < 0.05*, *p* < 0.01**, and *p* < 0.001***).

## Results

### Total phenolic and flavonoid contents in the LA extract

The Folin–Ciocalteu colorimetric assay indicated that the total phenolic content of the LA extract was 17.1 ± 1.2 µg GAE/g extract. The aluminum chloride-based colorimetric method revealed that the total flavonoid content in the LA extract was 51.5 ± 1.1 µg of the QE/g extract.

### UV-visible spectroscopy

**FIGURE 1:**
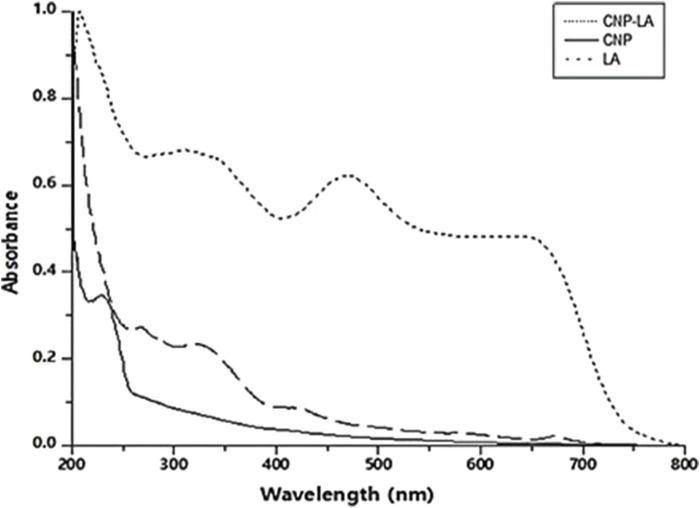
UV-visible spectrum (200–800 nm) of chitosan nanoparticles (CNP), *L. aspera* methanolic extract loaded within chitosan nanoparticles (CNP-LA), and *L. aspera* methanolic extract alone (LA).

The formation of CNP and CNP-LA was analyzed using the UV absorption characteristics. The UV–vis absorption spectra of CNP and CNP-LA are shown in Figure 1. The absorbance peaks for CNP and CNP-LA were at 249 nm and 279 nm, respectively. This is probably due to the adsorption/encapsulation of unsaturated molecules (aromatic/aliphatic) in CNP-LA. The absorbance of LA shows the difference in the absorption peaks due to the presence of phyto compounds. Other absorption bands at 418–510 nm, 250–370 nm, and 270–310 nm are the characteristics of alkaloids, flavonoids, and phenolic compounds, respectively.

### Particle size and zeta potential of CNP and CNP-LA

The particle size of the nanoparticles CNP and CNP-LA is in the range of 119–570 nm (Table 1). Furthermore, it was observed that the size of the nanoparticles was influenced by the concentration of the plant extract (LA). The zeta potential of CNP and CNP-LA is in the range of 19.4 ± 0.5 to 39.9 ± 0.3, indicating the highly cationic and stable nature of the nanoparticles.

**TABLE 1:** Physicochemical characteristics of both chitosan nanoparticles (CNP) and the *L. aspera* methanolic extract loaded within chitosan nanoparticles (CNP-LA) were evaluated at various TPP/extract weight ratios, with the chitosan concentration maintained constant throughout the experiments. The presented values indicate the mean ± standard deviation of replicates.

TPP (%)	Plant extract (%)	Particle size (nm)^a^	Zeta potential (mV)	PDI^a^	Encapsulation efficiency (%)^a^
0.5	-	442.5 ± 69.3^a^	36.2 ± 0.5	0.4 ± 0.1^b^	90.7 ± 2.5^a^
	1	369.0 ± 72.0	32.4 ± 1.2	1.8 ± 0.6	83.2 ± 0.5
	2	389.0 ± 68.6	31.7 ± 0.3	0.2 ± 0.3	93.6 ± 0.4
	5	375.7 ± 89.0	29.5 ± 1.9	1.2 ± 0.8	94.1 ± 3.5
	10	411.0 ± 24.0	27.1 ± 1.4	0.2 ± 0.2	90 ± 2.2
1	-	250.0 ± 53.0^b^	39.9 ± 0.3	1.1 ± 2.7^c^	92.6 ± 4.4^b^
	1	401.9 ± 76.7	32.7 ± 0.7	3.8 ± 3.1	98.6 ± 0.3
	2	440.9 ± 89.9	22.1 ± 0.5	1.4 ± 0.4	92.5 ± 0.0
	5	525.0 ± 79.7	19.8 ± 0.1	0.1 ± 0.0	90.4 ± 0.9
	10	570.0 ± 34.7	19.4 ± 0.5	0.7 ± 0.7	90.2 ± 0.7
2	-	119.7 ± 12.4^c^	32.4 ± 0.9	3.7 ± 2.2^c^	92.7 ± 0.0^c^
	1	218.4 ± 41.0	44.6 ± 1.2	0.4 ± 0.0	82.8 ± 3.8
	2	235.3 ± 23.4	34.5 ± 1.2	0.8 ± 0.6	71.8 ± 0.6
	5	258.6 ± 16.2	29.0 ± 0.2	2.7 ± 0.5	95.1 ± 0.1
	10	397.7 ± 76.5	26.4 ± 0.3	0.5 ± 0.6	95.4 ± 0.2

^a^ (*p* < 0.001), b (*p* < 0.01), and c (*p* < 0.05).

### Fourier-transform infrared spectroscopy of chitosan nanoparticles

**FIGURE 2:**
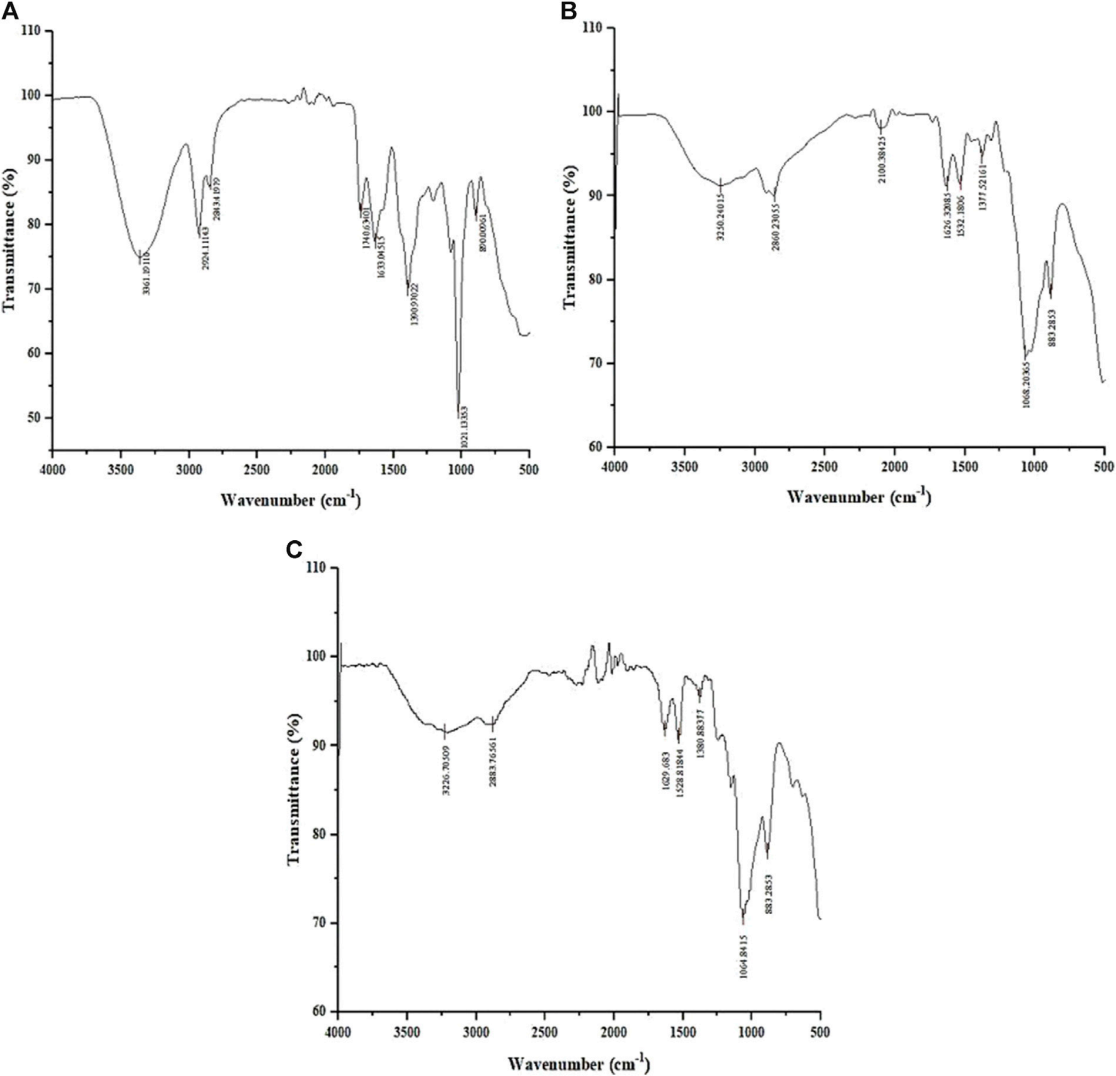
Fourier-transform infrared (FTIR) spectrum of **(A)** methanolic *L. aspera* extract (LA), **(B)** chitosan nanoparticles (CNPs), and **(C)** *L. aspera* methanolic extract loaded within chitosan nanoparticles (CNP-LA).

The FTIR spectrum of the *L. aspera* (LA) extract (Figure 2A) showed peaks at 3,361 cm^-1^, indicating the presence of primary and secondary amines and amides and a peak at 1,636 cm^-1^, indicating the existence of aromatic compounds. The peak at 1,309 cm^-1^ specifies the presence of nitro compounds, and the peak at 1,021 cm^-1^ indicates the presence of aliphatic amines. The peak at 860 cm^-1^ indicates the presence of alkyl halides, which is not present in the chitosan and CNP-LA spectra.

The FTIR spectra of CNP (Figure 2B) and CNP-LA (Figure 2C) showed bands at 3,250 and 3,226 cm^-1^, indicating the presence of hydroxyls in the chitosan nanoparticles. The peak at 3,279 cm^-1^ indicates the presence of alkynes (terminal), and peaks at 2,800–2,883 cm^-1^ and 883 cm^-1^ indicate the presence of alkanes and alkenes, respectively. The shortening of peaks at 3,410 cm^-1^, 3,371 cm^-1^, and 3,317 cm^-1^ indicates the presence of primary, secondary amines, and amides in CNP-LA, respectively.

## FESEM

**FIGURE 3:**
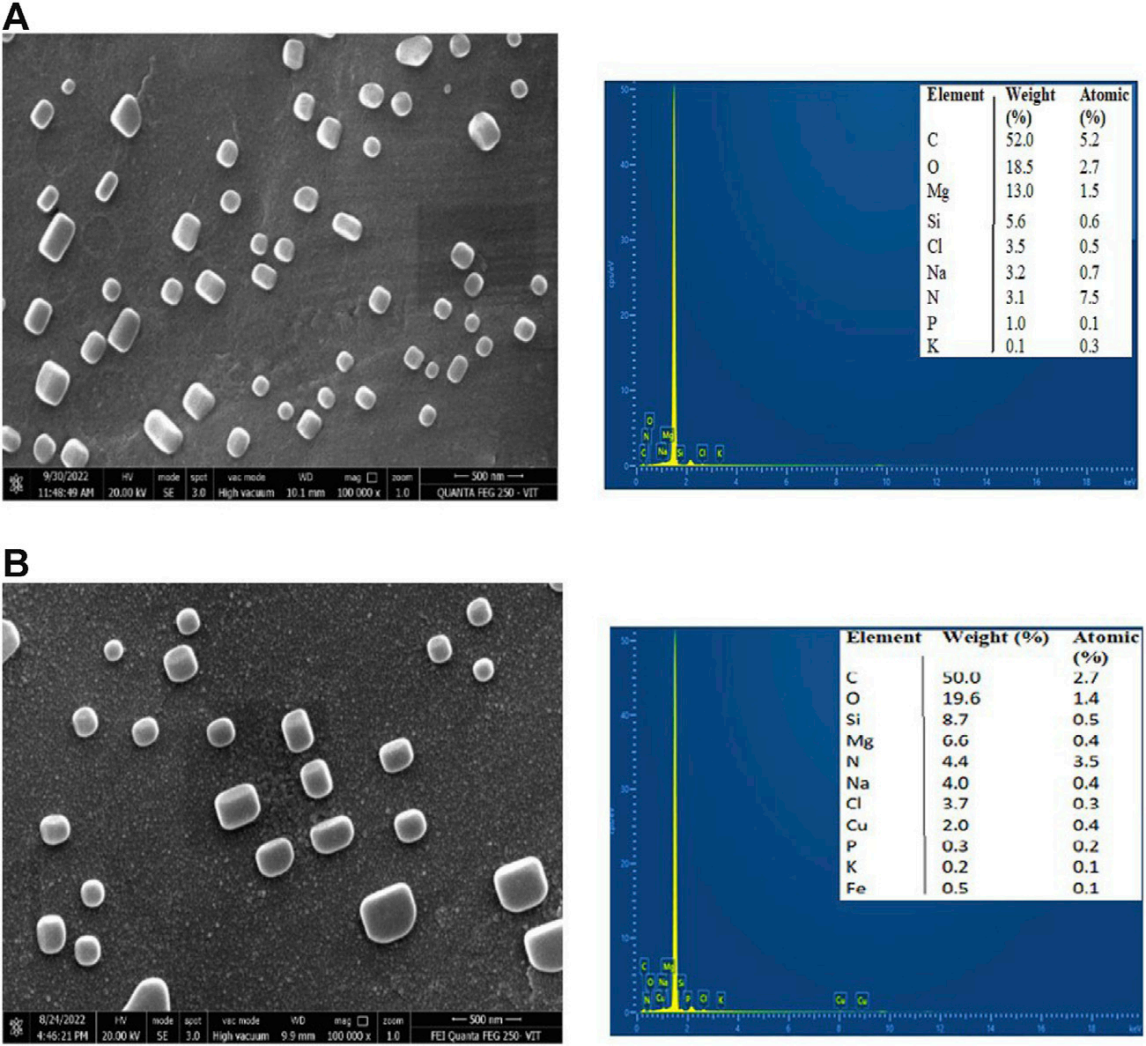
Field emission scanning electron microscopy (FESEM) images with EDX of **(A)** chitosan nanoparticles and **(B)** *L. aspera* methanolic extract loaded within chitosan nanoparticles (CNP-LA).

The surface morphology of CNP and CNP-LA was examined using FESEM (Figure 3). FESEM analysis showed that with the increasing concentration of LA, the size of the nanoparticles increases. Therefore, the size of CNP and CNP-LA differs depending on the concentrations of the loaded LA extract and is in the range of 119–570 nm. Both CNP and CNP-LA are of homogeneous size, and the nanoparticles were smooth, uniform, and spherical, without any trace of clumping or agglomeration.

The elemental composition and percentage of elements present in CNP and CNP-LA are given in Figure 3. The elements present in free CNPs are as follows: carbon 52.0%, oxygen 18.5%, magnesium 13.0%, silicon 5.6%, chlorine 3.5%, sodium 3.2%, nitrogen 3.1%, phosphorus 1.0%, and potassium 0.1%, while the elements present in extract-loaded chitosan nanoparticles (CNP-LA) are carbon 50.5%, oxygen 19.6%, silicon 8.7%, magnesium 6.6%, nitrogen 4.4%, sodium 4.0%, chlorine 3.7%, copper 2.0%, phosphorus 0.3%, potassium 0.3%, and iron 0.1%.

### Powder X-ray diffraction

**FIGURE 4:**
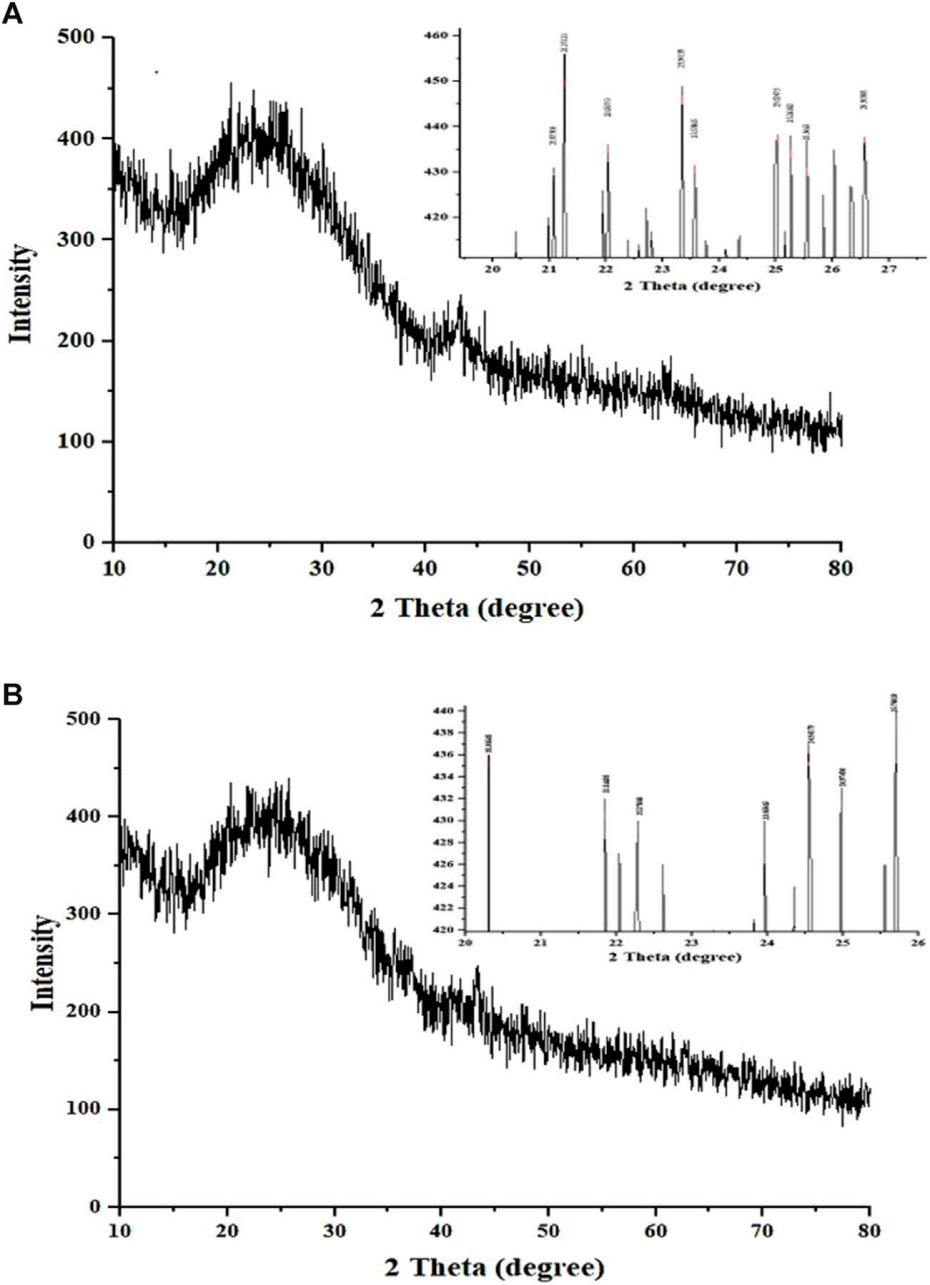
X-ray diffractogram of **(A)** chitosan nanoparticles and **(B)** *L. aspera* methanolic extract loaded within chitosan nanoparticles. The inset is the expanded view of the diffractogram in the given 2*θ* range.

The XRD patterns of CNP-LA were obtained and compared with those of CNP (Figure 4). The X-ray diffractogram of CNP showed several high-angle diffraction peaks at the following 2-theta values: 12.01°, 20.66°, 26.19° 29.82°, and 43.31°; CNP-LA showed several other high-angle diffraction peaks at 2-theta values: 11.23°,14.78°, 18.58°, 29.48°, 31.72°, 36.57°, 41.32°, and 43.14°. Free CNPs show less noticeable peaks with a very low intensity in the diffractogram while showing a complex network structure of interpenetrating polymer chains of chitosan cross-linked with one another by TPP counter ions. More noticeable peaks with high intensity for loaded chitosan nanoparticle CNP-LA can be seen.

### The release of LA extracts from CNP-LA (*in vitro*)

**FIGURE 5:**
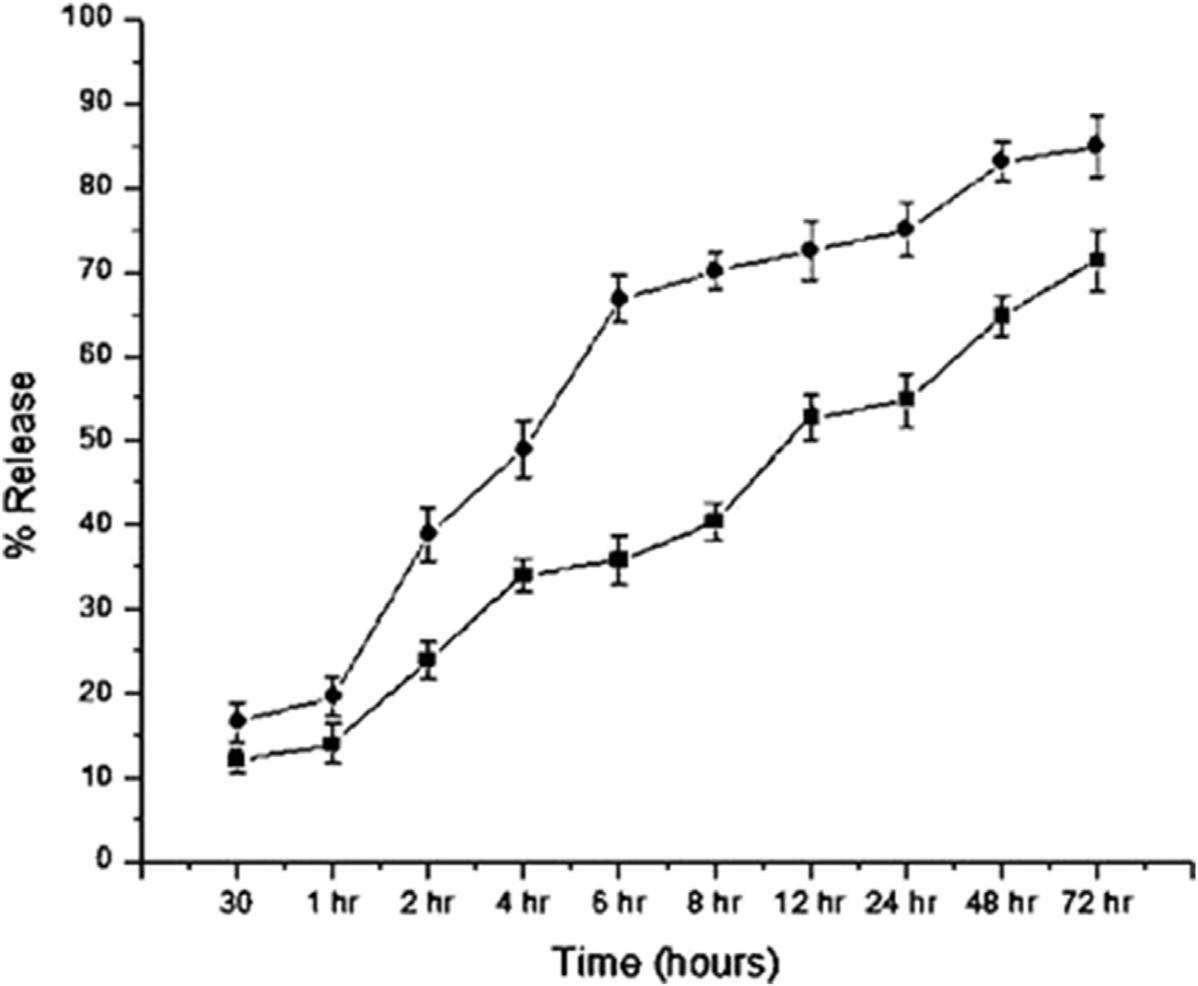
*In vitro* release profiles of the *L. aspera* methanolic extract loaded within chitosan nanoparticles (CNP-LA) plotted as a function of percentage release over the time at pH values 7.4 (circles) and 5.0 (rectangles). The values are represented as mean ± SD (n = 3).

For CNP-LA, the release profiles were investigated at two different pH values of 5.0 and 7.4, respectively (Figure 5). At a pH of 7.4, the maximum release at 72 h is 86.7%, and at a pH of 5.0, the maximum release at the same period is 73.10%. In the burst phase (first 30 min), 16.3% and 11.9% of the loaded extract are released at pH 7.4 and 5.0, respectively. However, in the subsequent 6 h, a more sustained release is observed at pH 5.0 (33.4%) than that at pH 7.4 (66.7%). The specific retention/release characteristics are derived from the molecular properties of the metabolites. It has to be noted that the release studies are only concerning the metabolites which absorb at 270 nm.

## Inhibition studies

### PLA₂ inhibition studies

**FIGURE 6:**
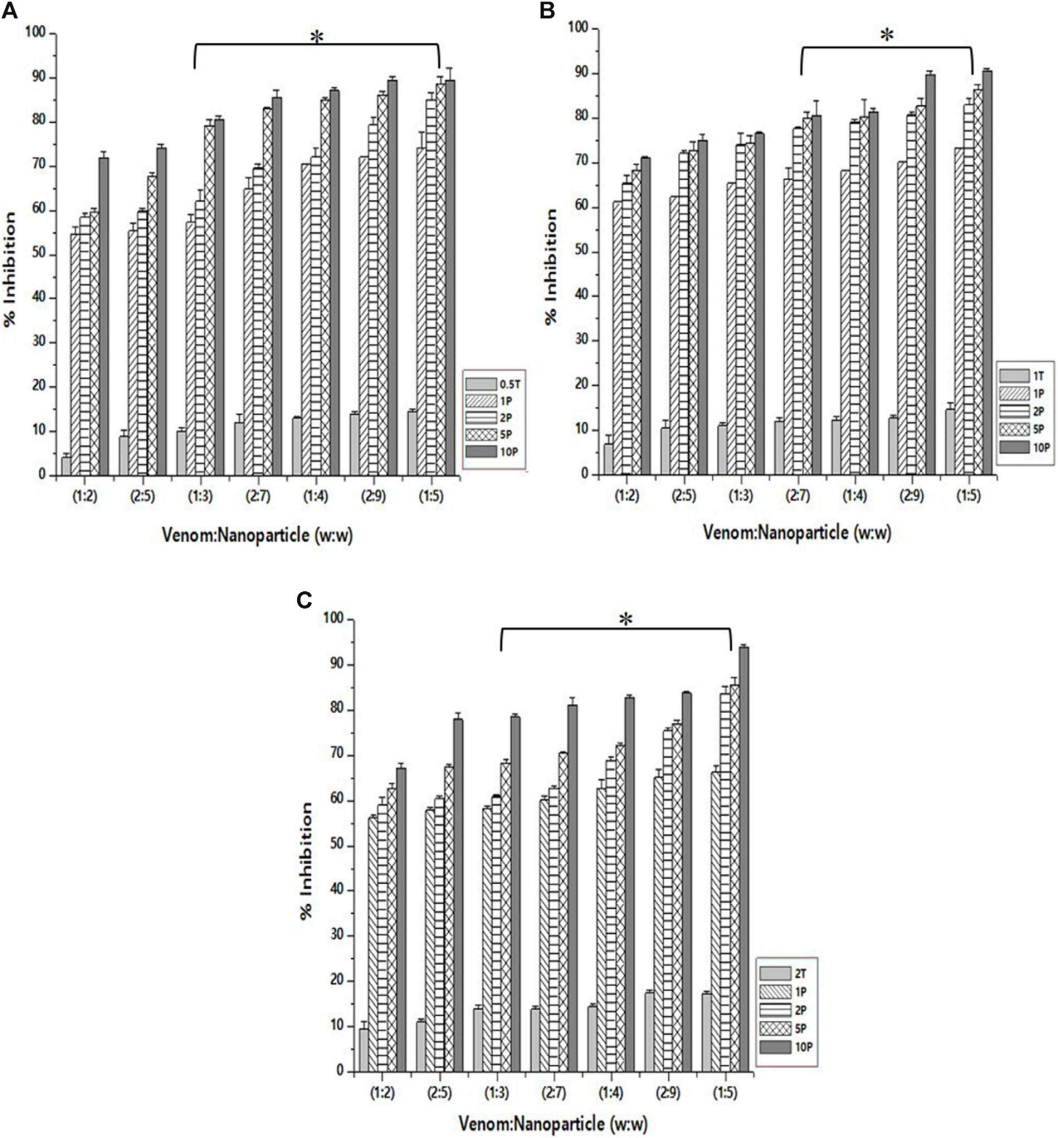
Effect of CNP and CNP-LA on *N. naja* venom phospholipase A₂ (PLA₂) was evaluated by pre-incubating with 50 µg of *N. naja* venom with different concentrations of chitosan nanoparticles (CNP) and the *L. aspera* methanolic extract loaded within chitosan nanoparticles (CNP-LA) at **(A)** 0.5 TPP, **(B)** 1 TPP, and **(C)** 2 TPP at various ratios for 1 h at 37°C. Values represent the mean ± SD of replicates (*p* < 0.001). In the figure, 2T represents the free nanoparticle, whereas 1P, 2P, 5P, and 10P represent the nanoparticles loaded within the plant extract.

The inhibition of PLA₂ activity varied for different nanoparticle formulations. This is obvious as they differ in size and, therefore, the amount of the encapsulated *L. aspera* methanolic extract. The maximum inhibition was observed with the venom: nanoparticle (V: N) ratio of 1:5 (Figure 6). For the nanoparticles prepared using 0.5%, 1.0%, and 2% TPP, the maximum inhibition was observed to be 89.5%, 90.5%, and 93.9%, respectively. It was observed that the free nanoparticle, even in the absence of the loaded plant extract, inhibited the activity of snake venom PLA₂ up to 17.3%. However, by increasing the amount of CNP-LA, the neutralization of *N. naja* venom progressively increases as the concentration increases in a dose-dependent manner. Antivenom was used as a qualitative reference, and the observed inhibition was 12.01%.

### Hemolytic inhibition assay

**FIGURE 7:**
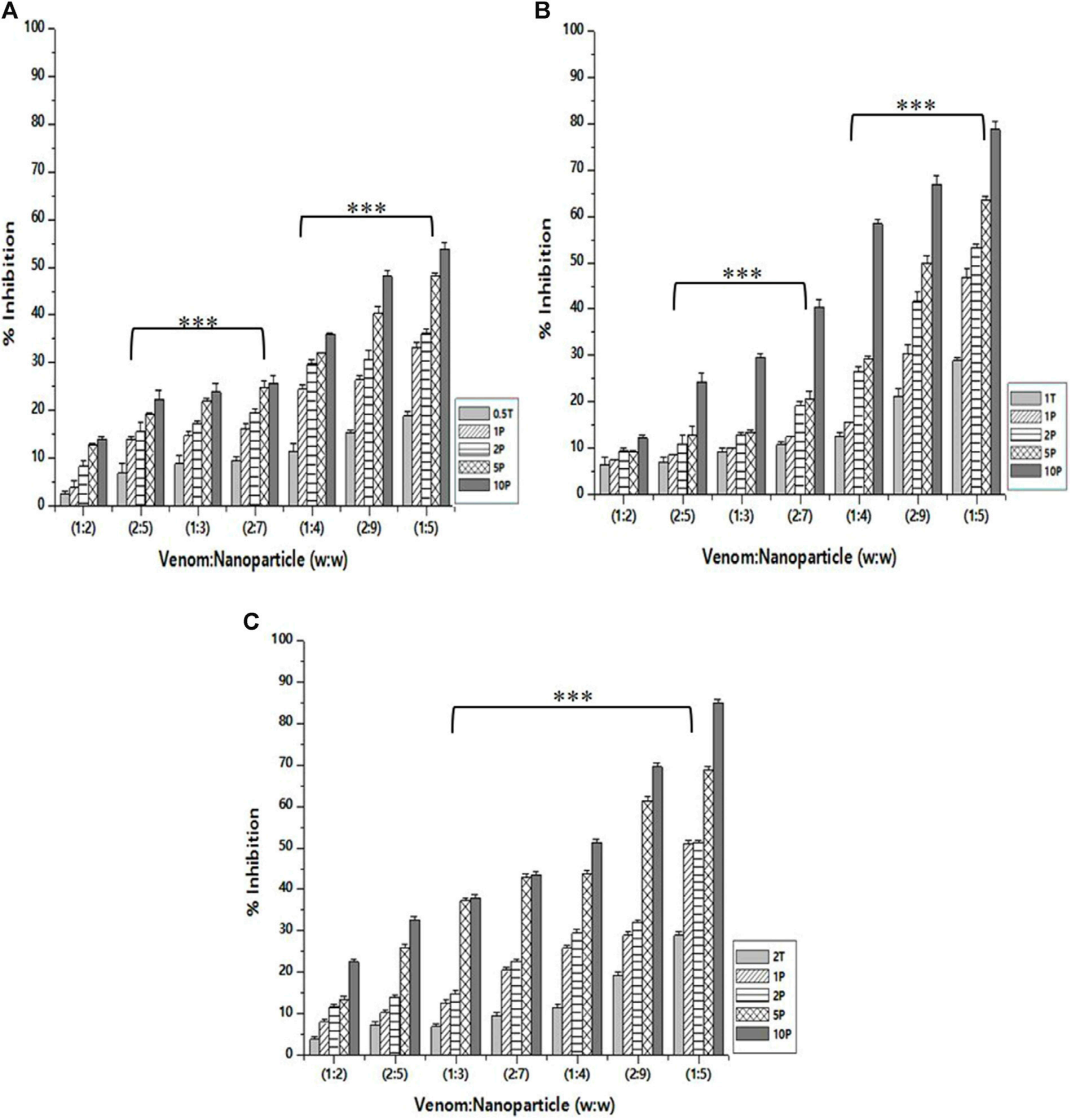
Effect of CNP and CNP-LA on *N. naja* venom hemolytic inhibition was evaluated by pre-incubating with 50 µg of *N. naja* venom with different concentrations of chitosan nanoparticles (CNP) and the *L. aspera* methanolic extract loaded within chitosan nanoparticles (CNP-LA) at **(A)** 0.5 TPP, **(B)** 1 TPP, and **(C)** 2 TPP at various ratios for 1 h at 37°C. Values represent the mean ± SD of replicates (*p* < 0.001). In the figure, 2T represents the free nanoparticle, whereas 1P, 2P, 5P, and 10P represent the nanoparticles loaded within the plant extract.

As in the case of PLA₂ inhibition activity, the venom: nanoparticle (V: N) ratio of 1:5 exhibited the maximum inhibition of hemolytic activity (Figure 7). For the nanoparticles prepared using 0.5%, 1.0%, and 2% of TPP, the maximum inhibition was observed to be 53.8%, 78.6%, and 84.9%, respectively. Compared to PLA₂ inhibition activity, there is a substantial difference in the hemolytic inhibition by the nanoparticle loaded with the extract. Additionally, the nanoparticle alone is capable of inhibiting up to 28.8% of the hemolytic activity of the venom. Antivenom used as a qualitative reference inhibited the venom hemolytic activity by 12.6%.

### Caseinolytic inhibition assay

**FIGURE 8:**
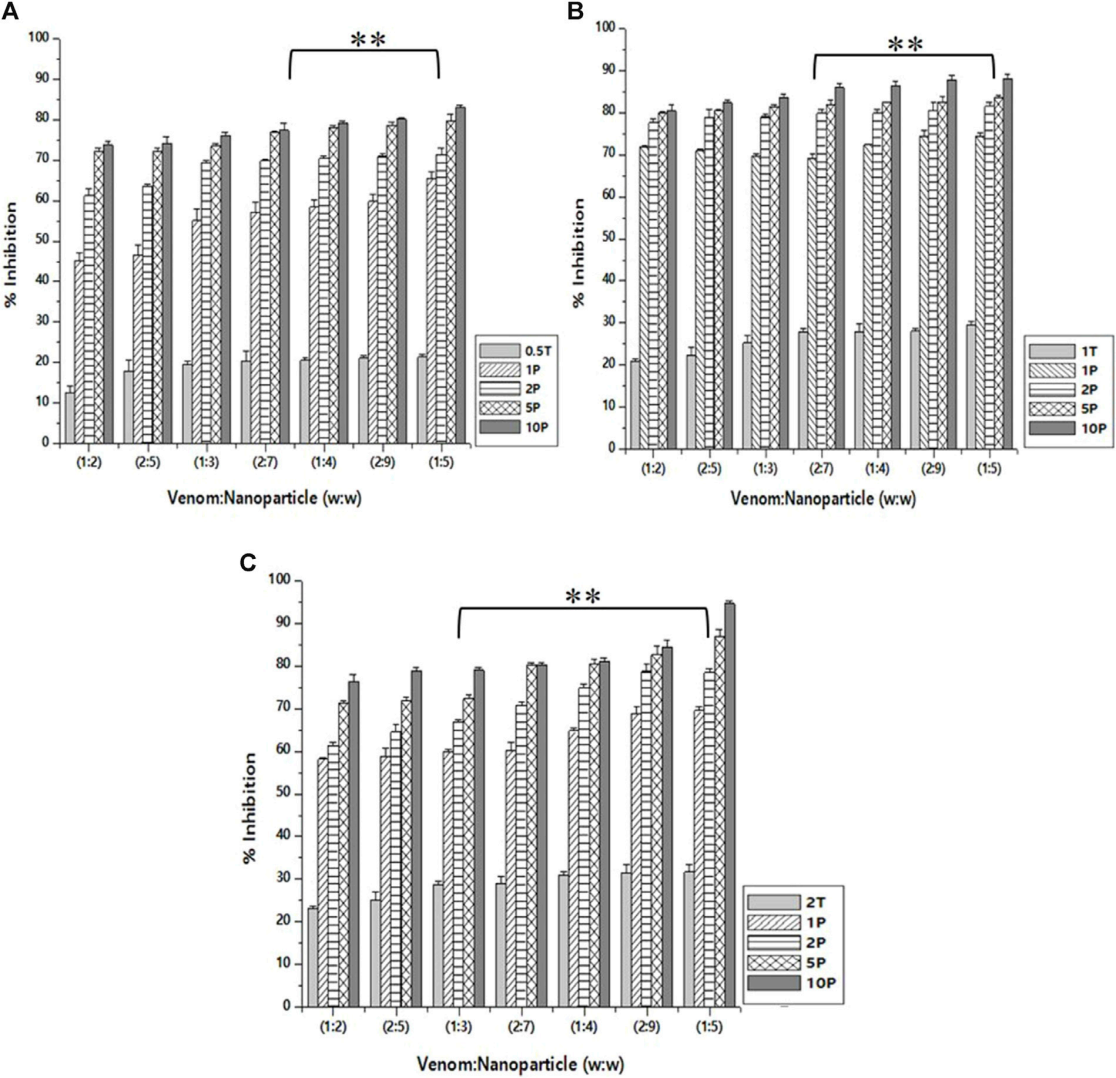
Effect of CNP and CNP-LA on *N. naja* venom caseinolytic inhibition was evaluated by pre-incubating with 50 µg of *N. naja* venom with different concentrations of chitosan nanoparticles (CNP) and the *L. aspera* methanolic extract loaded within chitosan nanoparticles (CNP-LA) at **(A)** 0.5 TPP, **(B)** 1 TPP, and **(C)** 2 TPP at different ratios for 1 h at 37°C. Values represent the mean ± SD of replicates (*p* < 0.01). In the figure, 2T represents the free nanoparticle, whereas 1P, 2P, 5P, and 10P represent the nanoparticles loaded within the plant extract.

As in the case of inhibition activity reported above, the venom: nanoparticle (V: N) ratio of 1:5 exhibited the maximum inhibition of caseinolytic activity (Figure 8). For the nanoparticles prepared using 0.5%, 1.0%, and 2.0% of TPP, the maximum inhibition was observed to be 83.2%, 87.6%, and 94.5%, respectively. The potential to inhibit the caseinolytic activity is similar to that of PLA₂ inhibition activity. Additionally, the nanoparticle alone is capable of inhibiting up to 31.6% of the caseinolytic activity of the venom. Increasing the concentration of the extract and the STPP in the formulation of nanoparticles, there is a significant increase in the inhibitory effect of CNP and CNP-LA against the venom for caseinolytic activity. Antivenom used as a qualitative reference exhibited 12.67% inhibition of caseinolytic activity.

## Discussion

Polyphenols and flavonoids are primary antioxidants and play a crucial role in scavenging free radicals (Rahman et al., 2006). Phenol-based antioxidants effectively regulate redox homeostasis during oxidative stress, contribute to cellular function regulation, and reduce the risk of chronic diseases (Vauzour et al., 2010). Therefore, it is essential to determine the total phenolic content in plant extracts. The phenolic content estimated in this study aligns with the previously reported values (Gopi et al., 2014), indicating that the LA extract is abundant in phenolic and flavonoid compounds, which are significant contributors to oxidative defense.

The absorption spectrum for chitosan nanoparticles showed maximum absorption of approximately 200–300 nm, as reported by Serrano (2013), Vaezifar et al. (2013), and Oh et al. (2019). The peak at 226–312 nm was identified as flavonoids and their derivatives. Two peaks at 404 and 462 nm are due to the presence of tannins, which have characteristic absorption peaks in the wavelength range of 350–500 nm (Bulla et al., 2021).

Hydrodynamic dimensions and surface charge play an important role in deciding the characteristics of particles. DLS and zeta potential provide information on the nanoparticle dispersion, size, and net charge. It has been reported that by varying the concentration of chitosan solutions and other parameters such as temperature, pH of the solution, and stirring duration, the particle size and the PDI (polydispersity index) for the chitosan nanoparticles are different (Khan et al., 2016; Rasaee et al., 2016; Thandapani et al., 2017; Agarwal et al., 2018; Iswanti et al., 2019). When CS concentrations increased, there is an increased availability of protonated amine groups for ionic gelation, which indicates the increase in the particle size. For the lowest doses of STPP and CS, the minimum size of 119 nm was achieved (Koukaras et al., 2012). The size of the nanoparticles was 340 nm at a CS/TPP w/w ratio of 4:1, but the size of the nanoparticles tends to increase at other CS/TPP ratios (Zhang et al., 2004). These results align with what is typically seen in chitosan nanoparticles produced through the ionic gelation method (Bugnicourt et al., 2014; Hamdan et al., 2023).

The elevated positive zeta potential is attributed to the presence of amino groups on the chitosan particle surface, diminishing the likelihood of aggregation and thereby enhancing the nanoparticle stability. The incorporation of the surfactant Tween 80 also influenced the nanoparticle size. Despite the introduction of anionic TPP to chitosan during fabrication, the resulting nanoparticles exhibited an overall positive charge, possibly due to the substantial molecular weight of chitosan (Silva et al., 2012; Miladi et al., 2015).

The analysis of the FTIR data in the nanoparticle formulations reveals the presence of various functional group peaks, indicating the interaction between the amino groups of chitosan and the phosphate groups of STPP (sodium tripolyphosphate), confirming the fabrication of the nanoparticles. This finding is consistent with a previous study conducted by Khan et al. (2016). Similar reports of chitosan nanoparticle formation with TPP (tripolyphosphate) ratios have also been documented (Qi et al., 2004; Bhumkar and Pokharkar, 2006). The peak at 564 cm^-1^ corresponds to the out-of-plane bending of NH and the out-of-plane bending of C−O (Varma and Vasudevan, 2020). The peaks between 1,000 and 1,050 cm^-1^ indicate the stretching vibrations of C–OH and C–O–C. The peak at 1,412 cm^-1^ represents C–N stretching vibrations (amide III band) (Uzun and Topal, 2013), while the peak at 1,568 cm^-1^ corresponds to –NH_2_ bending vibration (Rajam et al., 2011). The peak at 1,634 cm^-1^ is associated with the amide I group. The absorbance peak at 1879 cm^-1^, related to C–O and C–C stretching, indicates the compounds containing aldehyde, ketone, organic acid, or alkene groups (Liu et al., 2016). The peak at 2,360 cm^-1^ is due to C=O bending vibrations (Praffulla and Bubbly, 2018), while the peak at 2,934 cm^-1^ is attributed to aromatic C–H bending vibration (Kunasekaran and Krishnamoorthy, 2015). The peak at 3,429 cm^-1^ results from the O–H stretching (Kumirska et al., 2010), and the peaks observed between 3,400 and 3,800 cm^-1^ are attributed to O–H and NH_2_ bending vibrations, as well as intramolecular hydrogen bonding (Damiri et al., 2020). The peak at 3,881 cm^-1^ is associated with the O–H stretching in carboxylic acids (Anandalakshmi et al., 2016).

The size observed in DLS corroborates with the FESEM results. In the present study, the size of the nanoparticles formulated in the absence and presence of the extract was measured. The encapsulated LA extract altered the surface structure and particle size of chitosan nanoparticles. The average particle size of the nanoparticle is large (260 nm) in the encapsulated form compared to the extract-free nanoparticle (119 nm). Similar observations are reported in the literature. SEM images reported in earlier studies revealed spherical morphology and uniform size distribution in both chitosan nanoparticles (CSNPs) alone and those encapsulating pomegranate peel extract (PPE). The size of the pomegranate peel extract-loaded chitosan nanoparticle is larger (127.3 nm) than that of the extract-free particle (90.6 nm) (Soltanzadeh et al., 2021). Similarly, TEM analysis revealed that chitosan nanoparticles (CNPs) exhibited spherical particles ranging in size from 9 to 25 nm, while chitosan nanoparticles (CNPs) loaded with the ethanol extract of *Capparis cartilaginea* (CNPs/Cc) displayed spherical shapes with a size range of 18–30.1 nm (Salman et al., 2023). It is envisaged that the loading extract could create pores or voids on the interior of chitosan nanoparticles, potentially resulting in an overall increase in particle size. The increase in the size of the nanoparticle encapsulated with the molecules, especially the crude extracts, is because the entrapment occurs by occlusion, and therefore, molecules of diverse chemical nature will be encapsulated, which results in all types of intermolecular interactions, both stabilizing and destabilizing. Similar characteristics were observed in the chitosan nanoparticle preparations, as reported previously by Luque-Alcaraz et al. (2016). According to several investigations, CNPs have a good dispersion in the size of the nanoparticles and are entangled to form a larger exposed surface area, which makes CNPs appropriate for adsorption. The morphology of the nanoparticles depends on various parameters and adopts spherical (Mohammed et al., 2017), oval (Corazzari et al., 2015), or rod-shaped (Sivakami et al., 2013) structures. In terms of morphology, the chitosan nanoparticles (CSNPs) prepared in this study exhibited a spherical shape, consistent with observations in prior research (Yang et al., 2009). Both the free chitosan nanoparticles and the chitosan nanoparticles loaded with EPE displayed a uniform distribution without any noticeable agglomeration, maintaining a spherical size of approximately 50 nm (Egil et al., 2020).

The elements present in CNPs include nitrogen, oxygen, carbon, sodium, and phosphorus. The physical interaction between TPP and chitosan nanoparticles confirms the presence of phosphorus, which is in concurrence with the previous reports (MubarakAli et al., 2018). The diffraction pattern of free CNPs displayed a broad hump, consistent with findings in similar studies that showed various diffraction peaks at 2 theta = 20, indicative of the typical identification for semi-crystalline chitosan (Author Anonymous, 2013) and CNP-LA extending over a large range of 2 theta, suggesting that CS is present in the crystalline state. Another study has reported that a weak diffraction pattern was observed at peak 2θ = 10° (Rasaee et al., 2016) and a strong diffraction characteristic peak at 2θ = 20°, for the chitosan solution, therefore revealing the high degree of crystallinity nature for the chitosan. A predominant peak (110) for CS with a large diffraction peak at 2θ = 20° was also reported (Anusha and Fleming, 2016). From the diffraction spectrum for the CS and chitosan nanoparticles, diffraction peaks of 10.18° and 20.26° were observed at 2*θ*, respectively. Thus, it confirms the presence of the crystalline nature of the particle present in the synthesized chitosan (Budi et al., 2020). Chitosan’s diffraction pattern has peaks at 2*θ* = 9.28° and 20.18°, respectively, showing its crystalline form II, and a broad band was observed at 2*θ* = 30° (Divya et al., 2017). The broader peaks (or the width at half-height) are due to the size of the chitosan nanoparticles, which is in congruence with the particle size observed in FESEM. The crystalline nature of the nanoparticles is different in the free and plant extract-loaded nanoparticles.

The sustained release of the encapsulated plant extract was observed after an initial burst release at both the pH (5.0 and 7.4). The difference is due to the changes in the protonation states of both CS and TPP. As the formation of nanoparticles is attributed mainly to the intermolecular electrostatic interactions, the pH dependence in the stability and, therefore, the release of the encapsulated extract is expected. Both the polymers, chitosan and TPP, used to formulate the nanoparticle are charged polymers, and therefore, pH influences the formation, stability, and porosity of the nanoparticle. The pKa of chitosan amine is 6.3, and the STPP has pH-dependent ionization behavior, owing to different pKa values (0.9, 1.9, 5.3, and 7.7) (Pati et al., 2011). Under acidic conditions (pH 5.0, which is less than the pKa), chitosan amino groups are protonated. Therefore, both highly acidic (neutralizes the charge on TPP) and near neutral pH (neutralizes charges on chitosan) will destabilize the nanoparticle and result in the fragile interaction between chitosan and TPP. This is also reflected in the higher nanoparticle sizes at these pH conditions. The same is true for the plant extract-loaded nanoparticles. This is evident from the higher release of the encapsulated plant extract at neutral pH (7.0) than that at acidic pH (5.0). The release profile follows non-Fickian diffusion, where both diffusion and polymer relaxation contribute to the release of encapsulated molecules (Yao and Weiyuan, 2011). In addition to the pH, the molecular size and other physico-chemical properties of chitosan influences the size, porosity, and stability of the nanoparticles. Previous studies have also highlighted the influence of pH on the characteristics of chitosan nanoparticles, such as particle size, surface charge, porosity, stability, and the release of the encapsulated molecules. As indicated previously, the particles are more compact at pH near 5.0 and swell while deviating from these pH conditions. The size of *Rosmarinus officinalis* ethanolic extract-chitosan nanoparticle (ROEE-CNPs) is 48.80 ± 6.84 nm at pH 5.0 (Kasem et al., 2024). Chitosan nanoparticle-loaded grape extract pH adjusted between 3 and 4, and the particle size was 177.5 ± 2.12 nm (Soleymanfallah et al., 2022). CNPs produced using an aqueous extract of *Eucalyptus globulus* Labill leaves have the smallest particle sizes (with a size range between 6.92 and 10.10 nm) at 4.8 ± 0.02 (El-Naggar et al., 2022). The average size of CNP-LA nanoparticles reported in this study is 260 nm at pH 5.0. *Jatropha pelargoniifolia* extract-loaded chitosan nanoparticles exhibited different release profiles at pH 2.0 and pH 7.4. Within the initial 2 h, only 12% of the JP extract was released at pH 2.0, whereas at pH 7.4, the release was 25% (Alqahtani et al., 2021). The study reported that the mitomycin C-loaded chitosan nanoparticle cumulative release at two different pH values of 6.0 and 7.4 is 47% and 53%, respectively, at the end of 1,440 min (Kavaz et al., 2017). These results indicate the potential usability of CNP-LA for sustained-release applications, which can be controlled by the difference in the local pH conditions.

Snake venom phospholipase A₂ (PLA₂) is known to elicit a diverse array of pharmacological effects, including both pro- and anti-effects on myotoxicity, neurotoxicity, and cytotoxicity (Kini, 2003; Janardhan et al., 2019). PLA₂ is a multifunctional enzyme, and thus, exploring potential compounds for developing snake venom inhibitors involves a critical examination of PLA₂ activity inhibition. *Aristolochia radix* has been demonstrated to inhibit PLA₂ derived from *V. russelli* venom in a dosage-dependent manner (Sakthivel et al., 2013). Enhancing the efficiency of neutralizing snake venom PLA₂ is achieved through soy protein nanoparticles conjugated with anti-snake venom immunoglobulins (F (ab′)2 fragments) (Renu et al., 2014). Inhibiting *N. naja* snake venom PLA₂ activity using biosynthesized soy protein nanoparticles (SNP) from *Dryopteri cochleata* (at 0.1 mg/mL) significantly neutralizes *N. naja* venom activity. The neutralization potential of the biosynthesized SNP surpasses that of the whole-plant extract (Singh et al., 2020).

In addition to metalloproteases and phospholipases, Elapidae snake venoms are rich in cardiotoxins, cytotoxins, hemotoxins, and myotoxins, primarily associated with hemolysis and cytolysis (Dufton and Hider, 1988). Hemolysis is a characteristic phenomenon seen in cobra venoms due to the action of multiple components. Prior studies have shown that phospholipases play a role in breaking down the intact phospholipids found on the erythrocyte membrane, leading to hemolysis (Demel et al., 1975).

Serine proteases and metalloproteases are present in snake venom. These enzymes not only degrade the extracellular matrix but also disrupt the hemostatic system (Serrano, 2013). The primary role of snake venom proteases is to disrupt the general hemostatic pathway, leading to systemic hemorrhage. Snake venom metalloproteases, however, target a diverse range of substrates, including plasma proteins, membrane proteins, endothelial cells, proteins involved in platelet aggregation, and cells associated with the inflammatory response, ultimately resulting in severe hemorrhaging at the envenomation site (Fox and Serrano, 2009). *L. aspera* extracts have demonstrated the ability to decrease the proteolytic activity of snake venom, with previous research highlighting significant inhibitory effects, especially in the methanolic extract of *L. aspera* (Gopi et al., 2014). Elevating the concentration of plant extract-loaded nanoparticles effectively suppresses the proteolytic activity of the venom.

There have been a few reports on the formulation of nanoparticles to reduce the toxic effects of snake venom proteins. Silver nanoparticles prepared in the presence of *Alstonia scholaris* Linn bark extract effectively neutralized Viper russelli venom (Ghosh et al., 2021). Another report states that 2-hydroxy-4-methoxybenzoic acid (HMBA) from the root extract of the Indian Sarsaparilla (*Hemidesmus indicus*) has viper venom neutralizing effects in animal models (Gomes et al., 2016). A previous study reported that the encapsulation of *Naja naja oxiana* venom within chitosan nanoparticles presents potential as an alternative to traditional adjuvant systems (Mohammadpour Dounighi et al., 2012). *Cerastes cerastes* venom poly lactic-co-glycolic acid nanoparticles (Cc-PLGA NPs) provide protection against high lethal doses of viper venoms (Hamzaoui and Laraba-Djebari, 2021). Therefore, there is limited information on the application of nanoparticles in neutralizing the snake venom toxicity even though it offers novel avenues for venom neutralization and targeted therapy development. Nanoparticle-based approaches show promise in enhancing antivenom efficacy and mitigating venom-induced toxicity (Gomes et al., 2018). The investigation of CNP-LA against *Naja naja* venom toxicity makes a substantial contribution to the field of nanoparticle-based drug delivery systems for snakebite treatment. Such targeted intervention offers a promising avenue for more effective snakebite management, addressing critical aspects of venom toxicity and providing insights into developing safer and more efficient antivenom therapies. Therefore, our study significantly advances the understanding of nanoparticle-based drug delivery systems for snakebite treatment, offering a targeted and potentially safer approach to addressing venom-induced toxicity. To the best of our knowledge, this is the first report on the use of chitosan-based nanoparticle encapsulated with the *L. aspera* extract to neutralize the toxicity of Indian cobra venom.

Titanium dioxide nanoparticles (TiO_2_-NPs) neutralize *Daboia russelii* venom and *Naja kaouthia* venom (Chakrabartty et al., 2019). Silver nanoparticles, produced through the reduction of salts using a solid dispersion of curcumin (130 nm) and concentration of 0.081 mg mL^−1^), were utilized to counteract the toxic effects caused by *Philodryas olfersii* venom (Proença-Assunção et al., 2021). *Vitex negundo* gold nanoparticles neutralize *Naja kaouthia* venom toxicity-induced reactions in animal models (Gomes, 2015). However, metal-based nanoparticles can exhibit increased toxicity as their particle size decreases, leading to adverse effects on human health through mechanisms such as immunotoxicity, inflammation, oxidative stress, DNA damage, and cytokine inductions. Prolonged exposure to metal-based nanoparticles can have toxic effects on vital organs like the brain, liver, and kidney, raising concerns about their biocompatibility and potential harm to human health (Zhang et al., 2022). Therefore, there is a need for nanoparticles based on biopolymers. In the study conducted on the Indian spectacled cobra, the authors have detailed the formulation of the chitosan nanoparticle with snake venom to test the efficiency of the nanoparticle formulation (Mohammadpour Dounighi et al., 2012). In a previous study, the anti-ophidian properties of the *L. aspera* methanolic extract against the Indian cobra, *Naja naja*, venom enzymes were evaluated (Gopi et al., 2014). In continuation of our study, we have investigated the *L. aspera* methanolic extract loaded within chitosan nanoparticles. Chitosan is known for its biodegradability and non-toxic properties. CNPs exhibit a non-immunogenic nature, and their capacity to deliver therapeutic agents while reducing immune reactions is a valuable attribute in the field of nanomedicine (Mohammed et al., 2017). Chitosan nanoparticles (CNPs) provide a promising platform for drug delivery, minimizing toxicity, reducing immunogenic responses linked to antivenom use, and enhancing efficacy (Veronika and Peter, 2021). Therefore, the present study highlights that the encapsulation of herbal extracts into chitosan nanoparticles can mitigate the potential toxic effects of the extracts, rendering them safer for therapeutic applications. Chitosan nanoparticles facilitate the controlled release of herbal extract, thereby enabling sustained and prolonged therapeutic effects. The CNP-LA study represents an integration of nanotechnology with traditional medicine, presenting a hopeful direction for more efficient and safer snakebite treatments compared to other nanoparticle-based therapies.

## Conclusion and future perspective

The encapsulation of pharmaceutical compounds into chitosan nanoparticles is recognized as an effective strategy for enhancing sustained and controlled release. This study demonstrates the efficient encapsulation of the LA extract into CNPs, resulting in sustained release profiles at both pH 5.0 and 7.4. The encapsulated LA extract proved effective in inhibiting venom activities, including PLA₂, hemolytic, and proteolytic activities. Even though the study brings out the promising application of the chitosan-encapsulated plant extract for the management of snake venom toxicity, further animal models or clinical trials are essential to confirm the *in vivo* efficacy and safety. These studies will further provide insights into pharmacokinetics, biodistribution, and adverse reactions. Overall, the results provide useful insights into the use of CNP-LA as a promising herbal-based LA delivery system for the management of snakebites.

## Data Availability

The original contributions presented in the study are included in the article/Supplementary material; further inquiries can be directed to the corresponding author.

## Abbreviations

LA, *Leucas aspera;* CS, chitosan; CNP, chitosan nanoparticles; CNP-LA, chitosan nanoparticle-loaded *Leucas aspera* methanolic extract; PLA₂, phospholipase A₂.
